# Gas-Phase Anionic σ-Adduct (Trans)formations in Heteroaromatic Systems^1^

**DOI:** 10.1007/s13361-015-1122-1

**Published:** 2015-04-21

**Authors:** Magdalena Zimnicka, Witold Danikiewicz

**Affiliations:** Institute of Organic Chemistry, Polish Academy of Sciences, Kasprzaka 44/52, 01-224 Warsaw, Poland

**Keywords:** Gas-phase σ-adducts, Nitrothiophene, Nitrofuran, Ion-molecule reactions, Heteroaromatic carbanions

## Abstract

Anions of nitroderivatives of thiophene and furan were subjected to the reactions with selected C-H acids in the gas phase. Various structures and reaction pathways were proposed for the observed ionic products. In general, the reactions of heteroaromatic anions with C-H acids may be divided into three groups, depending on the proton affinity difference between C-H acid’s conjugate base and heteroaromatic anion (ΔPA). The proton transfer from C-H acid to heteroaromatic anion is a dominant process in the reactions for which ΔPA < 0 kcal mol^−1^, whereas the reactions with high ΔPA (ΔPA > 16 kcal mol^−1^) do not lead to any ionic products. The formation of σ-adducts and products of their further transformations according to the VNS, S_N_Ar, *cine*, and *tele* substitution mechanisms have been proposed for reactions with moderate ΔPA. The other possible mechanisms as S_N_2 reaction, nucleophilic addition to the cyano group, ring-opening pathway, and halogenophilic reaction have also been discussed to contribute in the reactions between heteroaromatic anions and C-H acids.

**Electronic supplementary material:**

The online version of this article (doi:10.1007/s13361-015-1122-1) contains supplementary material, which is available to authorized users.

**Graphical Abstract:**
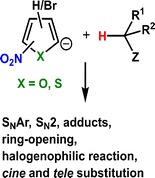
ᅟ

## Introduction

Anionic σ-adducts (often referred to as Jackson-Meisenheimer or Meisenheimer complexes) are formed as the result of addition of a nucleophile to electrophilic aromatic ring. In condensed phases this addition is well recognized for both aromatic and heteroaromatic systems and a wide range of products may be formed from further transformations of these adducts, depending on the reaction conditions and properties of substrates used in the reaction [1–5].

**Scheme 1:**
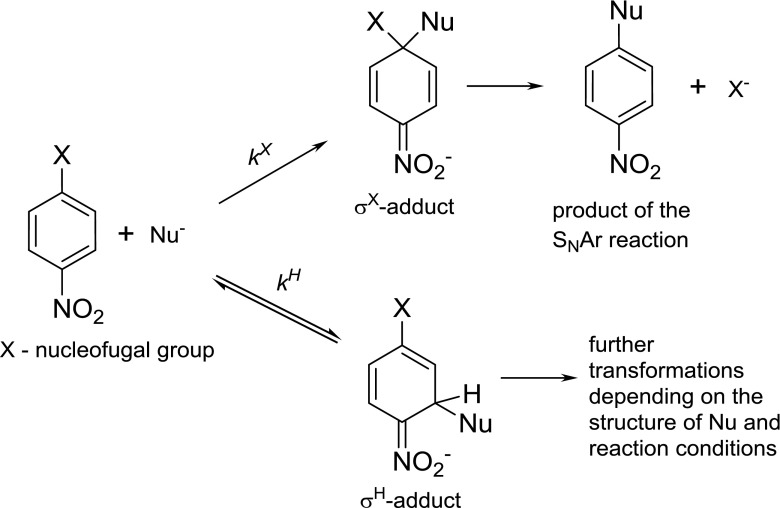
Formation and further transformations of σ^X^ and σ^H^-adducts in solution

An addition can occur only in the *ortho* and *para* position with respect to the nitro group because only such adducts are stabilized by resonance. In the case of a nitroaromatic ring that contains a substituent X either in the *ortho* or *para* position and that can act as a leaving group, two types of σ-adducts can be formed: σ^X^-adduct and σ^H^-adduct (Scheme 1). It is well documented that the formation of σ^X^-adducts is an irreversible process and this adduct is usually a transition state in the aromatic nucleophilic substitution reaction (S_N_Ar) [1]. The situation is different for the case of σ^H^-adducts, where formation is reversible and further transformations are strictly related to the type of nucleophile and reaction conditions. The direct conversion of the σ^H^-adduct into the product of nucleophilic substitution of hydrogen, via the addition-elimination pathway in which hydrogen anion is eliminated, is difficult because of the high energy barrier of C-H heterocyclic bond cleavage and is known only for some examples in condensed phase (i.e., for Chichibabin reaction [6]). Thus, the nucleophilic substitution of hydrogen is accomplished indirectly from the further transformations of σ^H^-adduct such as oxidative nucleophilic substitution of hydrogen (ONSH) [7], transformation into nitrosocompounds [8, 9], transformation of σ^H^-adduct according to ANRORC mechanism (addition of nucleophile - ring opening - ring closure) [10–12], vicarious nucleophilic substitution of hydrogen (VNS) [3, 4], *cine* and *tele* nucleophilic substitution of hydrogen [13].

It has to be stressed that the formation of σ^H^-adducts is a faster process than the formation of σ^X^-adducts. However, in the case when σ^H^-adduct cannot be transformed quickly to the hydrogen substitution products, only S_N_Ar reaction is observed. If the reaction conditions for further transformation of σ^H^-adducts are favorable, substitution of hydrogen is a dominating process even in the presence of very good leaving groups in the nitroaromatic ring [3–5]. Recently, we have proven in our group that the formation of σ^H^-adducts is a faster process compared with the formation of the σ^X^-adducts also in the gas phase [14].

In comparison to the exhaustive solution-phase studies, the number of publications describing the formation of σ-adducts and their further transformations in the gas phase is much lower. Especially the knowledge about the gas-phase σ-adduct formation in heteroaromatic systems is scant and it is limited to the one publication describing the reactivity of 1,3,5-triazine towards nucleophiles [15]. More information is available concerning the gas-phase σ-adducts of nitroarenes with nucleophilic reagent. In our lab, we have proven that the formation of stable gas-phase σ-adducts of nitroarenes with carbanions is possible [16, 17]. We have also observed the gas-phase S_N_Ar reactions of halonitroarenes with carbanions as well as some other transformations of σ-adducts [18–20]. Similar results were obtained by the Cooks’ group [21–24]. On the other hand, Chiavarino et al. proved by the IRMPD measurements accompanied by quantum chemical calculations that the products of the gas-phase addition of sufficiently basic anions to nitroarenes are indeed σ-adducts [25, 26]. However, the gas-phase reactions of nucleophiles with nitrothiophenes and nitrofurans have not been reported so far.

The presence of a heteroatom in an aromatic ring significantly affects the reactivity with nucleophilic agents. Significantly lower aromaticity of some heteroaromatic rings, such as of thiophene and furan, compared with the benzene ring (which causes a lower loss of stabilization energy on going from substrates to σ-adducts) [27, 28], near sp^3^ hybridization of carbon atoms in thiophene and furan (the same as is observed at the reaction center of σ-adduct formation) [29], improved ability of the π-orbitals of thiophene and furan aromatic systems to interact with electron-withdrawing substituents (i.e., the ability of the thiophene and furan rings to transmit the electronic effects of substituents more effectively than in benzene and involving the transmission also through heteroatoms), all these factors render the heteroaromatic systems of thiophene and furan more reactive versus nucleophilic agents with respect to benzene derivatives.

In this paper, we describe preliminary results of the generation and further transformations of the anionic σ-adducts of nitroderivatives of thiophene and furan with selected carbanions in the gas phase. The conditions required for the generation of adducts and plausible structures of their further transformations are considered based on the reactivity and specificity of heteroaromatic systems towards nucleophilic agents reported for solution phase, and by some simple proton affinity calculations.

## Experimental

**Scheme 2:**
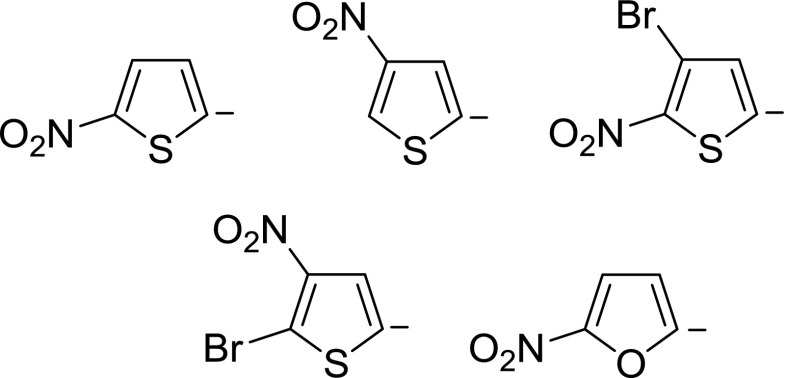
Nitrothiophene and nitrofuran anions studied in the present work

All measurements were performed using an API 365 triple quadrupole mass spectrometer (AB SCIEX, Framingham, MA 01701, USA) equipped with an electrospray ion source. The methodology for the investigation of gas-phase reactions was analogous to that previously described for studies of ion-molecule reactions between derivatives of phenide ions with C-H acids and is briefly outlined herein [16, 17]. The selected nitroheteroaromatic anions (Scheme 2) were formed in the ion source (medium pressure part) via decarboxylation of the appropriately substituted carboxylate anions. The ion source parameters were optimized in order to obtain the highest possible abundance of the nitroheteroaromatic anions. Selected with the first quadrupole, anions were subjected to the reactions with C-H acid admixed to the nitrogen introduced into the collision cell. The nominal cell voltage was set to −5 eV to suppress collision-induced dissociation (CID). The reaction products were then analyzed using the third quadrupole. Saturation of the curtain gas with the substrate vapors was used alternatively to allow the reaction to take place in the medium pressure section of the ESI ion source, hence enabling the CID of reaction products to be performed. Fragmentation spectra were recorded with nitrogen as a collision gas at various collision energy (CE) values, depending on the experiment. All CE values are reported in the laboratory frame.

Proton affinities (PA) at 298 K were calculated using the Gaussian 09 suite of programs [30]. Calculations were performed using a hybrid B3LYP functional on a B3LYP/6-311+G(3df,2p)//B3LYP/6-31G(d) level. Proton affinities of the C-H acid conjugate bases discussed in this manuscript were collected in Table S1 (Supporting Information). The experimental values for some of these anions were also given [31]. In the manuscript, we used calculated values of PA instead of the experimental ones. The exceptions were the proton affinities of Cl^¯^ and Br^¯^, for which the experimental values were used.

## Results and Discussion

### Generation and the Gas-Phase Stability Studies of Heteroaromatic Anions

In the first series of experiments, we examined the generation of selected nitroheteroaromatic anions from the appropriately substituted carboxylate anions via decarboxylation and their gas-phase stability, which in turn is highly required for further investigation of their ion-molecule reactions. The heteroaromatic anions should be stable enough to reach the collision cell, in which the gas-phase reactions with C-H acids occur, without any additional fragmentations. This is a very important factor since the reactions of selected anions, and not the reactions with the fragment ions, are being monitored. Another important factor is a well-defined position of a negative charge in heteroaromatic anions. The heteroaromatic anions (shown in Scheme 2) have no acidic hydrogen atoms, thus according to the earlier remarks on the isomerization of anions generated in the medium pressure part of an ESI ion source, the position of the negative charge should be determined by the position of the carboxylic group in the respective acids [16].

**Figure 1:**
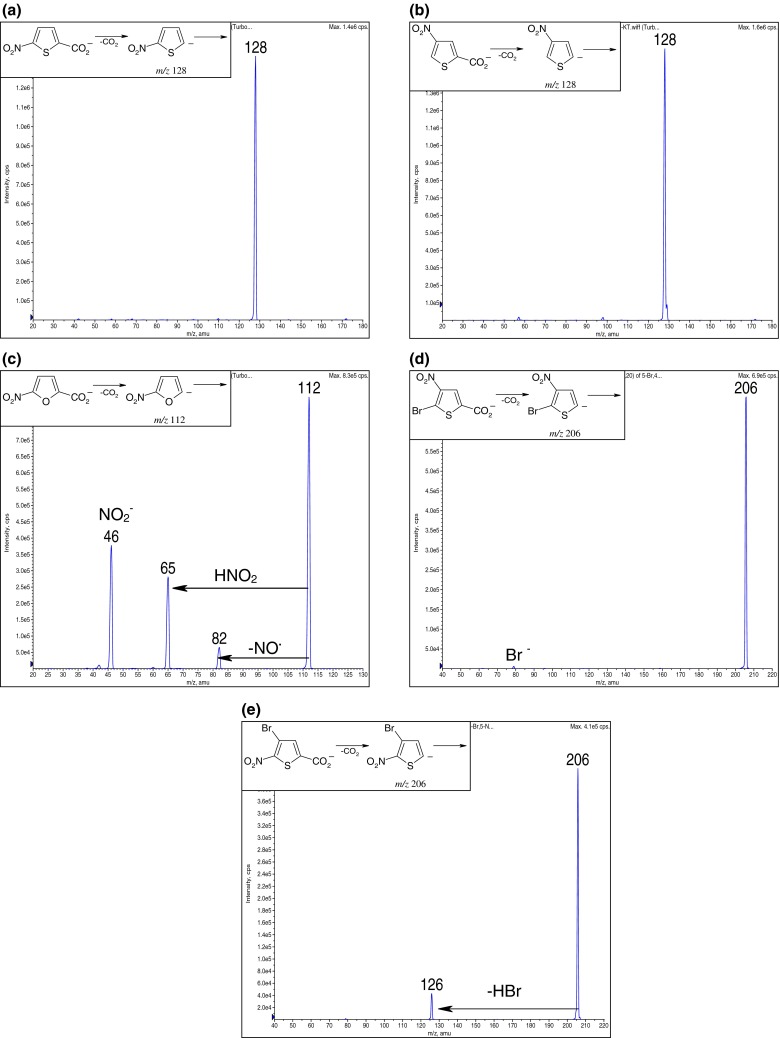
CID mass spectra (collision energy, CE = 5 eV) of heteroaromatic anions: **(a)** 2-nitrothiophene 5-anion, **(b)** 3-nitrothiophene 5-anion, **(c)** 2-nitrofuran 5-anion, **(d)** 2-bromo-3-nitrothiophene 5-anion, and **(e)** 3-bromo-2-nitrothiophene 5-anion

The results on the generation and stability of examined anions were obtained from the analysis of their CID spectra (Figure 1). The CID spectra were recorded at the lowest possible collision energy of 5 eV, the same as it was used in the gas-phase reaction experiments. Both isomeric nitrothiophene anions, i.e., 2-nitrothiophene 5-anion (Figure 1a) and 3-nitrothiophene 5-anion (Figure 1b), were very stable and no fragmentations were observed during the collision experiment. In contrast to the nitrothiophene anions, 2-nitrofuran 5-anion was unstable and even at the lowest collision energy dissociated by losing NO–, HNO_2_, and NO_2_^¯^ (Figure 1c). The fragmentation pathways leading to the indicated fragments of the nitrofuran anion may involve a ring opening. This process is highly probable taking into account the low aromaticity of the furan ring, which is 4.5 kcal mol^−1^ (according to the aromaticity scale based on the Dewar resonance energy, DRE), compared with the resonance energy of benzene (DRE_benzene_ = 22.6 kcal mol^−1^) [28]. Due to the low stability of 2-nitrofuran 5-anion, one may expect the additional reactions of ion fragments to occur.

The presence of a bromine atom in the heteroaromatic ring of nitrothiophene anions (Figure 1d and e) induces a slight decrease of stabilization as it was seen in the spectra by the presence of Br^¯^ (*m/z* 79) and ion resulting from the elimination of HBr (*m/z* 126). The presence of these additional peaks in the spectra of isomeric bromonitrothiophene 5-anions may also be assigned to a ring-opening pathway by the low resonance energy of thiophene ring, which is 6.5 kcal mol^−1^. It is worth noticing that the ion at *m/z* 126 may correspond to the formation of 3,4-didehydrothiophene anion. However, this process is not expected, since no experimental evidence of the existence of such type of hetaryne structures have been obtained, but one precedent, where the existence of 2,3-didehydrothiophene, formed in the flash-vacuum thermolysis of thiophene-2,3-dicaboxylic acid anhydride, was postulated [32].

### Reactions of Nitrothiophene Anions with C-H Acids

The results of the gas-phase reactions of 2-nitrothiophene 5-anion with selected C-H-acids are summarized in Table 1. The appropriate spectra are included in the Supporting Information Figure S1.

**Table 1:** Results of the Ion-Molecule Reactions of 2-Nitrothiophene 5-Anion with Selected C-H-acids

No.*	C-H acid [Y]	ΔPA	Reaction results				
			[A]^−^	[Y – H]^−^	[A – HNO_2_]^−^	[A – HCl]^−^	Other products
1.1	CHCl_3_	−4	-	+	-	-	-
1.2	ClCH_2_CN	−4	-	+	-	+	Cl^−^
1.3	ClCH_2_CO_2_Me	−2	-	+	-	+	Cl^−^, [A – MeOH]^−^
1.4	Cyclopentanone	5	+	-	+	n/a	-
1.5	CH_3_COCH_3_	7	+	-	+	n/a	-
1.6	CH_3_CO_2_Et	7	+	-	-	n/a	[A – EtOH]^−^
1.7	CH_3_CN	10	+	-	+	n/a	-
1.8	CH_2_Cl_2_	14	-	-	-	+	-

* No. = spectrum number in Supporting Information.

ΔPA = proton affinity difference between C-H acid conjugate base and 2-nitrothiophene 5-anion (calculated within this work as 362 kcal mol^−1^).

[A]^−^ = adduct.

[Y – H]^−^ = C-H acid anion resulting from the proton transfer.

[A – HNO_2_]^−^ = anion resulting from HNO_2_ elimination from adduct.

[A – HCl]^−^ = anion resulting from HCl elimination from adduct.

[A – ROH]^−^ = anion resulting from ROH (alcohol molecule) elimination from adduct.

In the reactions of 2-nitrothiophene 5-anion with C-H acids, the conjugate base of which has a lower PA than the 2-nitrophenide 5-anion (i.e., in the reactions with chloroform, methyl chloroacetate, and chloroacetonitrile), adduct formation was not observed. The main reaction products were the anions of the appropriate C-H acids resulting from the proton transfer with the formation of anion with the lower proton affinity. Additionally, the reactions with methyl chloroacetate and chloroacetonitrile led to the products corresponding to the formation of anions resulting from HCl elimination from adduct and Cl^¯^. In the case of the reaction with methyl chloroacetate the low intensity peak corresponding to the ionic product from MeOH elimination from adduct [A – MeOH]^¯^ was also observed. The product of this type was also detected in the reaction with ethyl acetate as [A – EtOH]^¯^ ion. The addition of a nucleophile to carbonyl group of ester followed by the elimination of an alcohol molecule (ester hydrolysis) is a well-known process in a gas phase [20, 33]; therefore, it will not be discussed in this work.

**Scheme 3:**
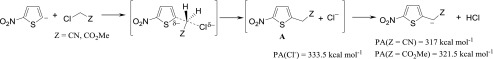
Reaction of 2-nitrothiophene 5-anion with chloroacetonitrile and methyl chloroacetate according to S_N_2 mechanism

The presence of [A – HCl]^¯^ ions may be explained by S_N_2 reaction with the mechanism’s details shown in Scheme 3. Taking into account the reactions in solution, Cl^¯^ ion as the S_N_2 reaction product should be observed. However, owing to the higher proton affinity of Cl^¯^ compared with the proton affinity of [5-nitro-2-thienylacetonitrile anion or methyl 2-(5-nitrothiophen-2-yl)ethanoate anion] [A – HCl]^¯^, the latter ion was postulated as the product of S_N_2 reaction.

The gas-phase nucleophilic displacement reactions were the subject of extensive studies mainly in saturated systems [34]. A variant of the nucleophilic substitution, i.e., halogenophilic reactions (also known as halophilic reactions) in which the nucleophile attacks the halogen instead of the carbon atom, which in this case acts as the leaving group, were also observed in aromatic systems [16, 35, 36]. The examples of the gas-phase reactions in which an arene carbanion initiates the addition process, e.g., reactions with carbonyl compounds and α,β-unsaturated compounds possessing electron-withdrawing groups have been also recently reported [19, 20, 37]. These studies show that indeed the arene carbanion may react as an effective nucleophile producing a broad set of products. Hence, the heteroaromatic carbanions may be also considered as active nucleophiles in addition processes.

**Scheme 4:**
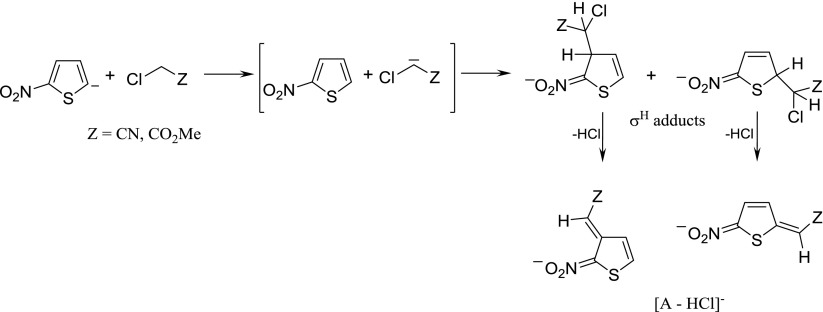
Reaction of 2-nitrothiophene 5-anion with chloroacetonitrile and methyl chloroacetate according to proton transfer followed by σ^H^-adduct formation and elimination of HCl

The other possible mechanism leading to the formation of [A – HCl]^¯^ may include proton transfer from C-H acid to 2-nitrophenide 5-anion, followed by the formation of σ^H^ adduct and HCl elimination from the adduct, according to the well-known VNS reaction (Scheme 4).

The results obtained so far do not allow us to unambiguously determine the exact mechanism of [A – HCl]^¯^ formation. The main question that should be answered is whether in the first step of the reaction the proton transfer from C-H acid to 2-nitrophenide 5-anion occurs. Taking into account the difference in the proton affinities between 2-nitrophenide 5-anion and C-H acid conjugate bases (Table 1) it can be assumed that the exothermic process of proton transfer will destabilize the complex of the reactants in the gas phase, causing its dissociation. However, it should be also noted that the exothermicity of this process is not too large (2–4 kcal mol^−1^); considering the difficulties in estimating the exact error of PA calculation, it could be even less. Therefore, one can expect that [A – HCl]^¯^ ionic products may be formed according to both of the abovementioned mechanisms.

Formation of Cl^¯^ in the reactions of 2-nitrophenide 5-anion with methyl chloroacetate and chloroacetonitrile may be explained by fragmentation of their conjugate bases. The fragmentation spectra revealed that such explanation may account for chloroacetonitrile. The most probable explanation of Cl^¯^ formation in the reaction with chloroacetate is a subsequent fragmentation of [A – MeOH]^¯^.

In the reactions of 2-nitrothiphene 5-anion with C-H acids, the conjugate base of which has a higher PA than the 2-nitrothiophene 5-anion, but not higher than the energy released during ion-molecule association in the gas phase, which is typically about 18 kcal mol^−1^ [38, 39], formation of adducts was mainly observed. The conceivable structures accessible for associates of heteroaromatic anion with C-H acid may correspond to σ-adducts, π complexes, or hydrogen-bonded complexes. However, considering the main requirement for adduct formation, which is the endothermic proton transfer from C-H acid to heteroaromatic anion (which allows the stabilization of the resulting ion–molecule complexes) [18] and further transformations of adducts, the structure of σ-adduct may be safely postulated. This is in agreement also with the results obtained by Chiavarino et al. [25] who proved the existence of anionic σ-adducts in the gas phase.

The exception in adduct formation is the reaction with dichloromethane, in which only the [A – HCl]^¯^ product (*m/z* 176) was observed. Similarly to the previous reactions as in this case, the [A – HCl]^¯^ may be formed according to the S_N_2 and/or VNS mechanisms.

**Scheme 5:**
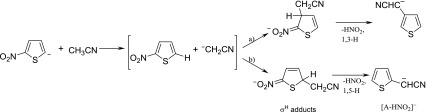
Reaction of 2-nitrothiophene 5-anion with acetonitrile

In the spectra recorded during the reactions of 2-nitrothiophene 5-anion with cyclopentanone, acetone, and acetonitrile the peaks corresponding to adducts were accompanied by the products of [A – HNO_2_]^¯^ resulting from HNO_2_ loss from an adduct. One of the possible explanations of the formation of [A – HNO_2_]^¯^ ions is the *cine* (pathway a) and/or *tele* (pathway b) substitution of the nitro group followed by proton transfer, as depicted in Scheme 5.

**Figure 2:**
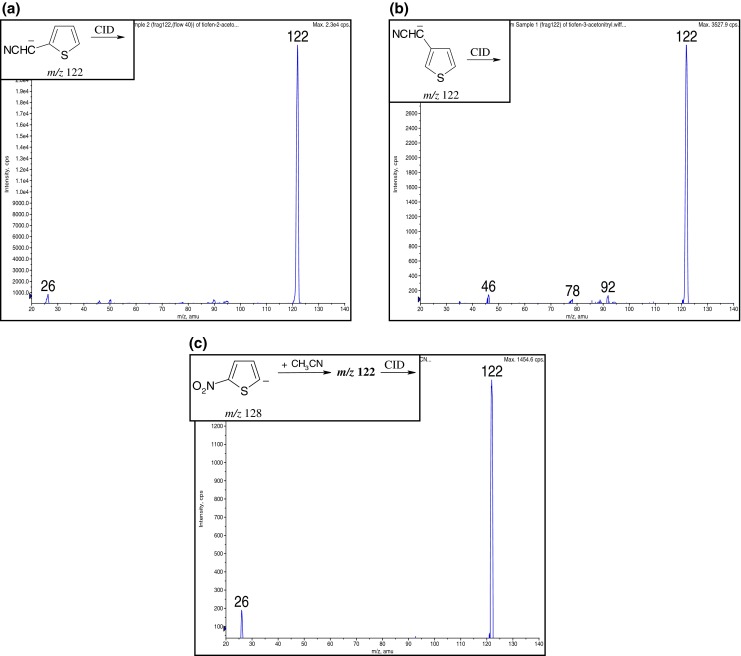
CID spectra (CE = 15 eV) of anions generated from deprotonation of **(a)** 2-thiophenoacetonitrile, **(b)** 3-thiophenoacetonitrile, and **(c)** [A – HNO_2_]^¯^ ion formed upon reaction of 2-nitrothiophene 5-anion with acetonitrile

The examination of structure proposed for [A – HNO_2_]^¯^ was accomplished by comparison of the fragmentation spectra (CID spectra) recorded for ionic products with the general formula of [A – HNO_2_]^¯^ generated from the gas-phase reaction of 2-nitrothiophene 5-anion with acetonitrile with those of the proposed model anions (Figure 2).

**Figure 3:**
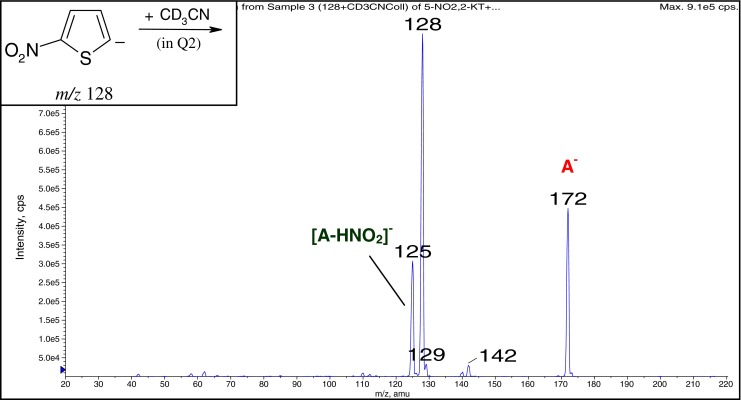
Gas-phase reaction of 2-nitrothiophene 5-anion with CD_3_CN

**Scheme 6:**
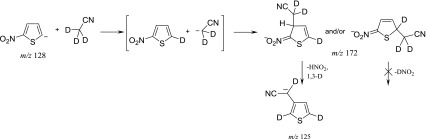
Proposed mechanism for the [A – HNO_2_]^¯^ ion formation in the reaction of 2-nitrothiophene 5-anion with CD_3_CN

The obtained results were inconclusive so additional experiment with deuterated acetonitrile (Figure 3) has been performed. It showed clearly that the formation of [A – HNO_2_]^¯^ ion according to the mechanism of the *tele* substitution of the nitro group has to be excluded because of the exclusive elimination of HNO_2_ instead of DNO_2_ from the adduct (Scheme 6). Therefore the *cine* mechanism for the [A – HNO_2_]^¯^ formation is expected. The formation of σ-adduct in position 3 of 2-nitrothiophene has been observed as the dominating or exclusive orientation in the vicarious nucleophilic substitution (VNS) of hydrogen in 2-nitrothiophene with various carbanions [40], however other reaction pathways, such as ring opening as a result of nucleophilic attack of the acetonitrile anion in position 5, cannot be excluded. Such reactions, in which the ring of 2-nitrothiophene is being opened, are known and postulated for the reactions occurring in solution with secondary amines [41].

Similar results to that found for 2-nitrothiophene 5-anion have been obtained for 3-nitrothiophene 5-anion with C-H acids (Table 2). In this case, according to the orientation in nucleophilic addition observed in solution [40], one can expect the formation of an adduct exclusively in position C2.

**Table 2:** Results of the Ion-Molecule Reactions of 3-Nitrothiophene Anion with selected C-H-Acids

No.*	C-H acid [Y]	ΔPA	Reaction results				
			[A]^−^	[Y – H]^−^	[A – HNO_2_]^−^	[A – HCl]^−^	Other products
2.1	CHCl_3_	−8	-	+	-	-	-
2.2	ClCH_2_CN	−8	-	+	-	+	Cl^−^
2.3	ClCH_2_CO_2_Me	−6	-	+	-	+	Cl^−^, [A – MeOH]^−^
2.4	Cyclopentanone	1	+	-	+	n/a	-
2.5	CH_3_COCH_3_	3	+	-	-	n/a	NO_2_^−^
2.6	CH_3_CO_2_C_2_H_5_	3	-	-	-	n/a	[A – EtOH]^−^
2.7	CH_3_CN	6	+	-	-	n/a	-
2.8	CH_2_Cl_2_	10	-	-	-	-	-

*No. = spectrum number in Supporting Information.

ΔPA = proton affinity difference between C-H acid conjugate base and 3-nitrothiophene 5-anion (calculated within this work as 366 kcal mol^−1^).

[A]^−^ = adduct.

[Y – H]^−^ = C-H acid anion resulting from the proton transfer.

[A – HNO_2_]^−^ = anion resulting from HNO_2_ elimination from adduct.

[A – HCl]^−^ = anion resulting from HCl elimination from adduct.

[A – ROH]^−^ = anion resulting from ROH (alcohol molecule) elimination from adduct.

**Scheme 7:**
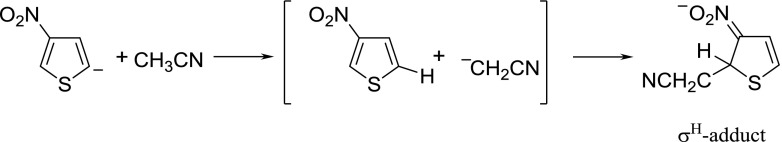
Formation of σ^H^-adduct in the reaction of 3-nitrothiphene 5-anion with acetonitrile

The results of the reactions of 3-nitrothiophene 5-anion with C-H acids, the conjugate base of which has a lower PA than the heteroaromatic anion, are strictly the same as those obtained for isomeric 2-nitrothiophene 5-anion; hence, the analogous explanation for the formation of [A – HCl]^¯^ and Cl^¯^ ions is postulated. In most cases, the reactions with C-H acids such as cyclopentanone, acetone, and acetonitrile lead to the formation of adducts. However, in these cases, the intensities of the peaks in the mass spectra corresponding to adducts were very low. Overall decrease of intensity of the adduct peaks as well as the lack or very low intensity of the peaks corresponding to the products of their further transformations indicated the lower reactivity of 3-nitrothiophene towards nucleophilic addition in comparison to 2-nitrothiophene. The observed decrease in reactivity can be explained by considering the mechanism of the adduct formation, taking acetonitrile as the example (Scheme 7).

The formation of σ^H^-adduct in position C2 requires an attack of the acetonitrile anion from above or below the heteroaromatic ring. Since the moving anion must overcome the repulsive energy derived from π electrons of the thiophene ring and the electron pair located at the sulfur atom, this transition requires energy. The calculations have shown that the activation energy of such a movement in the case of benzene ring is approximately 13 kcal mol^−1^ [42]. Taking into account the endothermicity of this transfer, it seems possible that the activation energy of such a transition is too high and, consequently, the corresponding adducts are formed at very low abundance or are not formed at all.

### Reactions of 2-Nitrofuran 5-Anion with C-H Acids

The results of the reactions of 2-nitrofuran 5-anion with selected C-H acids are summarized in Table 3. Because of the general similarity of the ionic products formed in the reactions of heteroaromatic anions with C-H acids, we would like to focus only on the differences induced by the presence of the oxygen atom in the aromatic ring.

**Table 3:** Results of the Ion-Molecule Reactions of 2-Nitrofuran Anion with Selected C-H-acids

No.*	C-H acid [Y]	ΔPA	Reaction results				
			[A]^−^	[Y – H]^−^	[A – HNO_2_]^−^	[A – HCl]^−^	Other products
3.1	CHCl_3_	−7	-	+	-	-	-
3.2	ClCH_2_CN	−7	-	+	-	-	Cl^−^
3.3	ClCH_2_CO_2_Me	−5	-	+	-	-	Cl^−^, [A – MeOH]^−^
3.4	cyclopentanone	2	+	+	-	n/a	-
3.5	CH_3_COCH_3_	4	+	+	-	n/a	[A – HNO]^−^
3.6	CH_3_CO_2_Et	4	+	-	-	n/a	[A – EtOH]^−^
3.7	CH_3_CN	7	+	-	+	n/a	[A – HNO]^−^
3.8	CH_2_Cl_2_	11	-	-	-	-	[A – NOCl]^−^

*No. = spectrum number in Supporting Information.

ΔPA = proton affinity difference between C-H acid conjugate base and 3-nitrofuran 5-anion (calculated within this work as 365 kcal mol^−1^).

[A]^−^ = adduct.

[Y – H]^−^ = C-H acid anion resulting from the proton transfer.

[A – HNO_2_]^−^ = anion resulting from HNO_2_ elimination from adduct.

[A – HCl]^−^ = anion resulting from HCl elimination from adduct.

[A – ROH]^−^ = anion resulting from ROH (alcohol molecule) elimination from adduct.

In contrast to the previous examined reactions, in this case the reaction of 2-nitrofuran 5-anion with cyclopentanone and acetone leads to the formation of appropriate anions of these C-H acids [Y – H]^¯^, despite unfavorable proton affinity difference. According to the earlier remarks, the stability of 2-nitrofuran 5-anion is very low and, hence, in the collision cell, where the ion-molecule reactions occur, the presence of the 2-nitrofuran 5-anion is accompanied by the ions derived from its fragmentation. Therefore, the presence of [Y – H]^¯^ may result from proton transfer from cyclopentanone or acetone to fragment anions such as anions resulting from HNO_2_ and/or NO radical loss from 2-nitrofuran 5-anion. Incorporation of NO_2_^¯^ in proton transfer process is not possible because of the lower proton affinity of NO_2_^¯^ (PA = 338.5 kcal mol^−1^) with respect to the proton affinities of cyclopentanone and acetone conjugate bases, which are 367 kcal mol^−1^ and 369 kcal mol^−1^, respectively. It is worth mentioning here that dehydrophenoxide radical anions derived from NO radical loss from nitrophenide anions readily react with C-H acids via proton transfer; hence, in the case of 2-nitrofuran 5-anion such a process seems to be also accessible. The final experimental evidence was obtained from the study of the reactions of anions resulting from HNO_2_ and NO radical loss from 2-nitrofuran 5-anion with cyclopentanone and acetone. Only the anion deriving from NO radical loss underwent the reaction with cyclopentanone and acetone. In addition, calculations have shown that indeed the proton affinity of such a radical-anion (this type of ions is also known as distonic anion containing both a radical and an ionic site on different atoms of the same molecule) is higher by 3 kcal mol^−1^ and 5 kcal mol^−1^ with respect to proton affinities of acetone and cyclopentanone anions.

In comparison to 2-nitrothiophene, the 2-nitrofuran seems to be less reactive in reactions with selected nucleophiles. This decrease in reactivity is due to the instability of 2-nitrofuran 5-anion in the experimental conditions as well as lower aromaticity of furan ring, which may facilitate ring-opening upon reactions with a nucleophile. It is worth noting that the decrease in reactivity associated with the ring-opening reaction of 2-nitrofuran has been observed in the reaction with some nucleophiles in the liquid phase [40].

### Reactions of Bromonitrothiophene Anions with C-H Acids

In the last series of experiments, we examined the reactions of bromonitrothiophene anions with C-H acids. Owing to the presence of a bromine atom in a heteroaromatic ring, we expected the S_N_Ar reaction to occur along with the reactions observed previously for nitrothiophene anions. The products of S_N_Ar reaction in the gas phase have been observed for halonitrophenide anions with selected C-H acids [18]. Taking into account the features of the heteroaromatic compounds (see the Introduction), one could anticipate that in this case the products of S_N_Ar reaction will be readily formed.

**Scheme 8:**
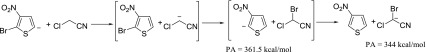
Proposed reaction pathway for the formation of bromochloroacetonitrile anion according to the halogenophilic mechanism

The results of the gas-phase reactions of 2-bromo-3-nitrothiophene 5-anion with selected C-H acids are summarized in Table 4. Similarly to the previously described reactions for unfavorable ΔPA value (i.e. ΔPA ≤ 0) the anions of the appropriate acids are formed along with [A – HCl]^¯^, [A – MeOH]^¯^, and Cl^¯^ ionic products, whereas the presence of low-intensity peak of Br^¯^ may be attributed to come from the fragmentation of the 2-bromo-3-nitrothiophene 5-anion (Figure 1d) or from decomposition of other fragment ions containing bromine atom. In the reactions with chloroacetonitrile and methyl chloroacetate, intensive peaks appear at *m/z* 152 and 185, corresponding to the formation of bromochloroacetonitrile anion and methylbromochloroacetate, respectively (structure assignment on the basis of the isotopic profiles). Reaction pathway leading to these ions is proposed to comprise the halogenophilic reaction followed by proton transfer according to the difference in proton affinities and formation of anion with lower proton affinity (Scheme 8).

**Table 4:** Results of the Ion-Molecule Reactions of 2-Bromo-3-Nitrothiophene 5-Anion with Selected C-H-acids

No.*	C-H acid [Y]	ΔPA	Reaction results				
			[A]^−^	[Y – H]^−^	Br^−^	[A – HCl]^−^	Other products
4.1	CHCl_3_	−2	-	+	-	-	-
4.2	Cl-CH_2_-CN	−2	-	+	+	+	Cl^−^, [A – HBr]^−^, *m/z* 152
4.3	Cl-CH_2_-CO_2_Me	0	-	+	+	+	[A – MeOH]^−^, *m/z* 185
4.4	Cyclopentanone	7	+	-	-	n/a	-
4.5	CH_3_-CO-CH_3_	9	+	-	+	n/a	-
4.6	MeCO_2_Et	9	-	-	-	n/a	[A – EtOH]^−^
4.7	CH_3_CN	12	+	-	-	n/a	
4.8	CH_2_Cl_2_	16	-	-	-	-	-

*No. = spectrum number in Supporting Information.

ΔPA = proton affinity difference between C-H acid conjugate base and 2-bromo-3-nitrothiophene 5-anion (calculated within this work as 360 kcal mol^−1^).

[A]^−^ = adduct.

[Y – H]^−^ = C-H acid anion resulting from the proton transfer.

[A – HBr]^−^ = anion resulting from HBr elimination from adduct.

[A – HCl]^−^ = anion resulting from HCl elimination from adduct.

[A – ROH]^−^ = anion resulting from ROH (alcohol molecule) elimination from adduct.

The presence of the products of halogenophilic reaction may be justified by the formation of a more stable 3-nitrothiophene 2-anion attributable to the Br^+^ detachment. Moreover, the reaction presented above is exothermic with the reaction enthalpy −17 kcal mol^−1^.

In the spectra recorded during the reactions of 2-bromo-3-nitrothiophene 5-anion with C-H acids for 0 < ΔPA < 16 kcal mol^−1^ the peaks corresponding to adduct formation are visible. Due to their very low intensity and the lack of products of their transformations, it is not possible to present a plausible explanation for their structure.

The results of the reactions of 3-bromo-2-nitrothiophene 5-anion with C-H acids are summarized in Table 5. In further discussion we focus only on the most important differences between reactions of isomeric bromonitrothiophene anions.

**Table 5:** Results of the Ion-Molecule Reactions of 3-Bromo-2-Nitrothiophene 5-Anion with Selected C-H-acids

No.*	C-H acid [Y]	ΔPA	Reaction results				
			[A]^−^	[Y – H]^−^	Br^−^	[A – HCl]^−^	Other products
5.1	CHCl_3_	2.5	+	+	-	+	[A - HNO_2_]^−^, [A - NOCl] ^−^
5.2	ClCH_2_CN	2.5	-	-	++	+	[A – HBr]^−^
5.3	ClCH_2_CO_2_Me	4.5	+	-	++	+	[A – MeOH]^−^
5.4	Cyclopentanone	11.5	+	-	+	n/a	-
5.5	CH_3_COCH_3_	13.5	+	-	+	n/a	-
5.6	MeCO_2_Et	13.5	+	-	-	n/a	[A – EtOH]^−^
5.7	CH_3_CN	16.5	-	-	-	n/a	-
5.8	CH_2_Cl_2_	21	-	-	-	-	-

*No. = spectrum number in Supporting Information.

ΔPA = proton affinity difference between C-H acid conjugate base and 3-bromo-2-nitrothiophene 5-anion (calculated within this work as 355.5 kcal mol^−1^).

[A]^−^ = adduct.

[Y – H]^−^ = C-H acid anion resulting from the proton transfer.

[A –HBr]^−^ = anion resulting from HBr elimination from adduct.

[A – HCl]^−^ = anion resulting from HCl elimination from adduct.

[A – ROH]^−^ = anion resulting from ROH (alcohol molecule) elimination from adduct.

**Scheme 9:**
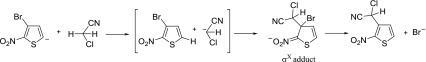
Proposed pathway for the reaction between 3-bromo-2-nitrothiophene 5-anion with chloroacetonitrile leading to the formation of Br^−^ anion

The formation of adducts and products of their further transformations was mostly observed for 2.5 ≤ ΔPA < 16.5 kcal mol^−1^. In contrast to the earlier discussed isomeric anion, in this case any peak corresponding to the product of halogenophilic reaction was not observed. Instead, the high-intensity peak corresponding to Br^¯^ was visible in the spectra recorded during the reactions with chloroacetonitrile, methylchloroacetate, cyclopentanone, and acetone. The proposed reaction pathway leading to the Br^¯^ formation is presented in Scheme 9, taking chloroacetonitrile as the example. It comprises the S_N_Ar reaction preceded by proton transfer. The final ionic product is Br^¯^ as the ion with lower PA (ΔPA = 3 kcal mol^−1^).

**Scheme 10:**
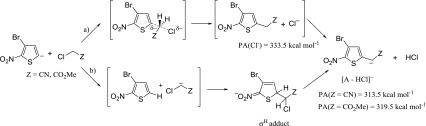
Proposed pathways for the reaction of 3-bromo-2-nitrothiphene 5-anion with chloroacetonitrile and methyl chloroacetate leading to the elimination of HCl molecule

In the reactions with chloroform, chloroacetonitrile, and methyl chloroacetate the [A – HCl]^¯^ is formed. This type of ionic product may result from S_N_2 reaction, according to the Scheme 10a. However, because of the favorable ΔPA between heteroaromatic anion and anion of the C-H acid conjugate base, in the first step of reaction the proton transfer from C-H acid to 3-bromo-2-nitrothiophene 5-anion may be privileged. A further step may consist of the formation of σ^X^ adduct (Scheme 9) and the formation of σ^H^ adduct (Scheme 10b). After elimination of HCl from the latter adduct the [A – HCl]^¯^ ionic product is formed. The proton transfer from HCl to [A – HCl]^¯^ is not expected due to the higher proton affinity of Cl^¯^ in comparison to [A – HCl]^¯^.

## Conclusions

In this manuscript, we reported preliminary results on the gas-phase ion-molecule reactions of electron-deficient heteroaromatic anions such as nitroderivatives of thiophene and furan with selected C-H acids. According to the previous gas-phase studies on σ-adduct formation and the features of heteroaromatic rings with respect to the nucleophilic agents, these reactions, under favorable conditions, [i.e., corrected difference between proton affinities of heteroaromatic anion and a C-H acid conjugate base (ΔPA)], were considered to lead to the formation of σ-adducts as well as to the products of their further transformations. Indeed, the results obtained so far clearly showed that under the correct value of ΔPA such adducts can be formed. This result was assumed by the observation of peaks with *m/z* values corresponding to the formation of adducts and products of their further transformations according to the VNS reaction, S_N_Ar reaction, and *cine* and *tele* nucleophilic substitution. However, for many of the reported reactions, it was not possible to clearly distinguish between products of the above-mentioned reactions and the products resulting from other possible mechanisms like S_N_2 reaction, nucleophilic addition to the cyano group, ring-opening pathway, etc. Therefore, further experimental and computational studies are needed to obtain a detailed picture on the gas-phase formation and transformation of the heteroaromatic σ-adducts. These studies are underway in this laboratory.

## Electronic supplementary material

Below is the link to the electronic supplementary material.

ESM 1 (DOCX 720 kb)
