# Supplementary material for: Gas-Phase Anionic σ-Adduct (Trans)formations in Heteroaromatic Systems1
Source: J Am Soc Mass Spectrom. 2015 Apr 21;26(7):1191–203. doi: 10.1007/s13361-015-1122-1 (PMC4475249; doi:10.1007/s13361-015-1122-1)
Supplement: Supplementary file 1 — (DOCX 720 kb) [file 13361_2015_1122_MOESM1_ESM.docx]

**Supporting Information**

Gas-Phase Anionic σ-Adduct (Trans)Formations in Heteroaromatic Systems

Magdalena Zimnicka, Witold Danikiewicz*

Institute of Organic Chemistry, Polish Academy of Sciences, Kasprzaka 44/52, 01-224 Warsaw, Poland

E-mails: [magdalena.zimnicka@icho.edu.pl](mailto:magdalena.zimnicka@icho.edu.pl), [witold.danikiewicz@icho.edu.pl](mailto:witold.danikiewicz@icho.edu.pl)

**Contents Page**

**Table S1.** Proton affinities of the anions discussed in this work. 2

**Figure S1.** Q3 mass spectra recorded for the products of the reactions of 2

2-nitrothiophene anion with C-H acids (referred to Table 1).

**Figure S2.** Q3 mass spectra recorded for the products of the reactions of 5

3-nitrothiophene anion with C-H acids (referred to Table 2).

**Figure S3.** Q3 mass spectra recorded for the products of the reactions of 8

2-nitrofuran anion with C-H acids (referred to Table 3).

**Figure S4.** Q3 mass spectra recorded for the products of the reactions of 11

3-bromo-2-nitrothiophene anion with C-H acids (referred to Table 4).

**Figure S5.** Q3 mass spectra recorded for the products of the reactions of 14

2-bromo-3-nitrothiophene anion with C-H acids (referred to Table 5).

**Table S1.** Proton affinities of the anions discussed in this work (kcal mol^-1^).

| **Anion** | **PA (calculated values)** | **PA (experimental values)** |
| --- | --- | --- |
| ¯CCl_3_ | 358 | 357.5 |
| ¯CH(Cl)CN | 358 | 357.5 |
| ¯CH(Cl)CO_2_Me | 360 | - |
| [cyclopentanone - H]¯ | 367 | 368 |
| ¯CH_2_COCH_3_ | 369 | 368 |
| ¯CH_2_CO_2_Et | 369 | 370.5 |
| ¯CH_2_CN | 372 | 373.5 |
| ¯CHCl_2_ | 376 | 375 |

**PA (calculated values) –** PA calculated using a hybrid B3LYP functional on a B3LYP/6 311+G(3df,2p) // B3LYP/6-31G(d) level;

**PA (experimental values) –** average experimental proton affinities taken from NIST database.

**Figure 1S.** Q3 mass spectra recorded for the products of the reactions of 2-nitrothiophene anion with C-H acids (referred to Table 1). In the figures, the peaks corresponding to the 2-nitrothiophene anion fragments are indicated (F symbol).

Figure 1.1

CCl_3_^-^

Figure 1.2

Cl^-^

ClCHCN^-^

**[A-HCl]^-^**

-NO**^.^**

**F**

**F**

**F**

Figure 1.3

Cl^-^

**[A-MeOH]^-^**

**[A-HCl]^-^**

Figure 1.4

**A^-^**

**[A-HNO_2_]^-^**

Figure 1.5

**A^-^**

**[A-HNO_2_]^-^**

[128+CO_2_]^-^

[186-NO^.^]^-.^

[128+CO_2_]¯

Figure 1.6

170

**A^-^**

**[A-EtOH]^-^**

Figure 1.7

**A^-^**

**[A-HNO_2_]^-^**

[169-NO^.^]^-.^

[128+CO_2_]^-^

[128+CO_2_]¯

Figure 1.8

**[A-HCl]^-^**

**F**

**Figure 2S.** Q3 mass spectra recorded for the products of the reactions of 3-nitrothiophene anion with C-H acids (referred to Table 2). In the figures, the peaks corresponding to the 3-nitrothiophene anion fragments are indicated (F symbol).

Figure 2.1

CCl_3_^-^

**F**

**F**

Figure 2.2

Cl^-^

ClCHCN^-^

**[A-HCl]^-^**

**F**

**F**

Figure 2.3

**[A-MeOH]^-^**

Cl^-^

**[A-HCl]^-^**

**F**

**F**

[128+CO_2_]¯

Figure 2.4

**A^-^**

x 20

**[A-HNO_2_]^-^**

**F**

**F**

Figure 2.5

**A^-^**

[128+CO_2_]^-^

NO_2_^-^

**F**

**F**

Figure 2.6

**[A-EtOH]^-^**

170

[128+CO_2_]^-^

**F**

[128+CO_2_]¯

Figure 2.7

**A^-^**

169

x 20

**F**

Figure 2.8

[128+CO_2_]^-^

**F**

**Figure 3S.** Q3 mass spectra recorded for the products of the reactions of 2-nitrofuran anion with C-H acids (referred to Table 3). In the figures, the peaks corresponding to the 2-nitrofuran anion fragments are indicated (F symbol).

Figure 3.1

CCl_3_^-^

**F**

**F**

NO_2_^−^

Figure 3.2

Cl^-^

ClCHCN^-^

**F**

**F**

NO_2_^−^

Figure 3.3

**[A-MeOH]^-^**

Cl^-^

**F**

**F**

NO_2_^−^

Figure 3.4

**A^-^**

[112+CO_2_]^-^

**F**

**F**

NO_2_^−^

[112+CO_2_]¯

Figure 3.5

**A^-^**

**[A-HNO]^-^**

**F**

**F**

NO_2_^−^

[112+CO_2_]¯

Figure 3.6

154

**A^-^**

**[A-EtOH]^-^**

**F**

**F**

NO_2_^−^

Figure 3.7

154

**A^-^**

**[A-HNO]^-^**

**[A-HNO_2_]^-^**

**F**

**F**

NO_2_^−^

[112+CO_2_]¯

Figure 3.8

**[A-NO-Cl]^-^**

**F**

**F**

NO_2_^−^

**Figure 4S.** Q3 mass spectra recorded for the products of the reactions of 2-bromo-3-nitrothiophene anion with C-H acids (referred to Table 4). In the figures, the peaks corresponding to the 2-bromo-3-nitrothiophene anion fragments are indicated (F symbol).

Figure 4.1

CCl_3_^-^

Br^-^

Figure 4.2

Br^-^

Cl^-^

**[A-HCl]^-^**

**[A-HBr]^-^**

[206+CO_2_]¯

Figure 4.3

Br^-^

**[A-HCl]^-^**

**[A-MeOH]^-^**

[206+CO_2_]¯

Figure 4.4

290

**A^-^**

x 20

**F**

Figure 4.5

Br^-^

**A^-^**

[206+CO_2_]^-^

Figure 4.6

[206+CO_2_]^-^

248

**[A-MeOH]^-^**

**F**

Figure 4.7

[206+CO_2_]^-^

**A^-^**

247

x 20

**F**

Figure 4.8

[206+CO_2_]^-^

**F**

**Figure 5S.** Q3 mass spectra recorded for the products of the reactions of 3-bromo-2-nitrothiophene anion with C-H acids (referred to Table 4). In the figures, the peaks corresponding to the 3-bromo-2-nitrothiophene anion fragments are indicated (F symbol).

Figure 5.1

CCl_3_^-^

**[A-HCl]^-^**

**A^-^**

277

[A-HNO_2_]^-^

[A-NOCl]^-^

**F**

Figure 5.2

Br^-^

**[A-HCl]^-^**

**[A-HBr]^-^**

**F**

Figure 5.3

**[A-MeOH]^-^**

282

Br^-^

**[A-HCl]^-^**

314

**A^-^**

**F**

Figure 5.4

[206+CO_2_]^-^

**A^-^**

Br^-^

**F**

Figure 5.5

[206+CO_2_]^-^

Br^-^

**A^-^**

**F**

Figure 5.6

248

294

[206+CO_2_]^-^

**[A-EtOH]^-^**

x 20

x 20

**A^-^**

**S**

**F**

Figure 5.7

[206+CO_2_]^-^

**F**

Figure 5.8

[206+CO_2_]^-^

**F**
